# Double Trouble: How Being Outnumbered and Negatively Stereotyped Threatens Career Outcomes of Women in STEM

**DOI:** 10.3389/fpsyg.2019.00150

**Published:** 2019-02-19

**Authors:** Ruth van Veelen, Belle Derks, Maaike Dorine Endedijk

**Affiliations:** ^1^Social, Health and Organizational Psychology Department, Faculty of Social Sciences, Utrecht University, Utrecht, Netherlands; ^2^Educational Sciences Department, Faculty of Behavioural, Management and Social Sciences, University of Twente, Enschede, Netherlands

**Keywords:** social identity threat, gender identification, masculine work contexts, gender (under)representation, work engagement, career confidence, Science Technology Engineering Math (STEM)

## Abstract

Masculine work contexts form an important source of social identity threat for working women. But what aspect of masculine work contexts is most threatening to women’s gender identity at work: A numerical majority of male colleagues (i.e., numerical male dominance), working in a profession in which women are negatively stereotyped (i.e., normative male dominance), or the combination? The current study aimed to disentangle these two aspects of masculine work contexts by testing its combined impact on the experience of gender identity threat among women and men who work in the STEM sector (i.e., Science, Technology, Engineering and Mathematics). A field study was conducted among women (*N* = 177) and men (*N* = 630) graduates holding an academic degree in a STEM educational program. Respondents either worked in- or outside the STEM sector (i.e., stronger vs. weaker gender stereotype) and estimated the ratio of men to women in their direct work environment. Results from a Structural Equation Model demonstrated that women in STEM face double trouble: The combination of working almost solely with male colleagues (being outnumbered) and working in the technical sector (where women are negatively stereotyped) predicted the highest levels of experienced gender identity threat, particularly among women who highly identified with their gender group. Gender identity threat, in turn, negatively predicted women’s work engagement and career confidence. Men did not face double trouble: Their experience of gender identity threat was not related to working in a masculine STEM sector. Importantly, considering that the women in this sample already hold a degree in STEM, and have proven their competence in STEM and resilience to gender stereotypes, this research reveals how in naturalistic work settings, prevailing social identity threats continue to affect women’s professional careers.

## Introduction

The STEM sector (i.e., Science, Technology, Engineering and Mathematics) is one of the most vital sectors for the economic competitiveness of European countries. With an academic degree in STEM, people have access to the largest number, the best-paying and fastest-developing jobs (Cedefop, 2016; European Union, 2016; European Commission, 2017). Yet the STEM sector remains a male dominated field; women are less likely than men to opt for STEM educational programs, to hold a degree in STEM, and to enter the labor force in the field of STEM (Hill et al., 2010; Catalyst, 2018). Recent statistics demonstrate that in the Netherlands – where the current study was situated – only 24% of STEM graduates are women. And of those women, a vast majority of 71% opts for a career *outside* STEM. As a result, a mere 13% of professionals in the STEM sector are women (Monitor Techniekpact, 2016). This puts the Netherlands at the bottom of European rankings in the share of women in STEM (Statistics Netherlands, 2016).

Although quite a number of studies have examined girls’ and women’s motivation to choose STEM as a field of study (e.g., Cheryan et al., 2009; Else-Quest et al., 2010; London et al., 2011; Thoman and Sansone, 2016), women holding a degree in STEM are a small and understudied group (but see Fouad et al., 2016). To our knowledge, there are no prior empirical studies that directly compare how women STEM graduates who opt for a career in the STEM sector experience working in a male dominated context, relative to those who opted out. The current paper aims to fill this gap in the literature and investigates how women’s own career perceptions are shaped by the fact that they work in-, or outside male-dominated STEM sectors.

Both in popular narrative (Sandberg, 2013; Kay and Shipman, 2014) and in scientific work (Hakim, 2000; Cech et al., 2011), it is often implied that women’s intrinsically lower levels of career confidence, motivation and ambition relative to men’s cause them to opt out of challenging careers in traditionally masculine STEM sectors. From this argument it would follow that women are just not that willing to ‘go the extra mile’ or to ‘make the sacrifices’ needed to succeed in these types of careers (Belkin, 2003). Indeed, gender differences in career confidence and ambition have been found in prior research (e.g., Van Vianen and Fischer, 2002; Cech et al., 2011). Yet we argue that these gender differences do not emerge in a social vacuum and that they are not always a matter of personal choice. Instead, we posit that gendered socio-cultural norms in STEM work contexts constrain women’s (more than men’s) career perceptions and impose barriers to building their career confidence and engagement in STEM (see also Peters et al., 2013).

This paper builds on social identity theory (SIT; Tajfel and Turner, 1979) to investigate what aspects of masculine work contexts may form career barriers among women STEM graduates. The SIT approach posits that in organizational contexts people’s attitudes and behaviors are determined, at least in part, by their group memberships (e.g., being a woman, a professional, a member of a team), and the importance people attach to these groups (Haslam et al., 2014). Specifically, we investigate how being a woman in a male dominated STEM sector may form a source of social identity threat (i.e., the feeling of being devalued or stigmatized at work on the basis of one’s gender identity; Tajfel and Turner, 1986), which may result in negative career-related outcomes such as lower work engagement and career confidence.

**FIGURE 1:**
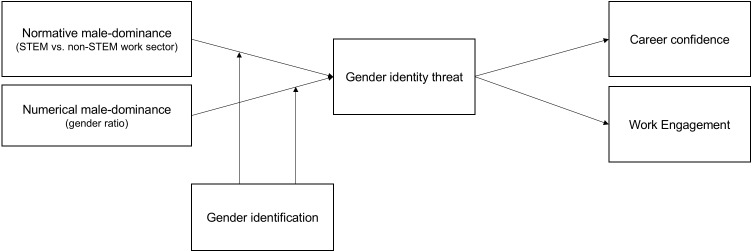
Conceptual model with hypothesized relationships.

In our investigation, we distinguish between two aspects that may signal institutional male dominance in work contexts (Gruber and Morgan, 2005). In addition, we investigate whether particularly women who strongly identify with their gender group experience strong gender identity threat in response to male dominant work contexts (Crocker and Major, 1989; Ellemers et al., 2002). The conceptual model is displayed in Figure 1 and tested among a sample of female and male STEM professionals. The inclusion and direct comparison of women to men allows us to test whether expected gender identity threat effects of male dominant work contexts indeed uniquely apply to women and thus form yet another explanation as to why particularly women in STEM tend to opt out.

## Theory and Hypotheses

### Social Identity Threat Among Women in STEM

Social identity threat is evoked when people feel concerned about being negatively treated, stereotyped or devalued in some way on the basis of their group membership (Tajfel and Turner, 1986; Crocker and Major, 1989; Branscombe et al., 1999). Gender identity threat as a specific form of social identity threat emerges when women or girls feel that they themselves as women, or their group as a whole is devalued or stigmatized. For example, when women feel judged based on their gender rather than their professional competence, or when women feel uncomfortable in work situations because of their gender, such as in relation to sexist remarks or jokes.

A large body of research has been devoted to gender identity threat and the conditions under which it is triggered. For example, women STEM students who watched a video about an engineering conference reported lower belonging and lower desire to participate in the conference when the men in the video were overrepresented compared to when the gender composition was equal (Murphy et al., 2007). Similarly, women confronted with gender stereotype-confirming commercials reported lower interest in educational and vocational options that involved technical domains, and avoided math tasks in favor of verbal tasks (Davies et al., 2002). Moreover, in terms of performance strategies, when women performed a task on which they were told that men perform better than women, they tended to focus on not failing on the task rather than being successful, especially when they had to perform the task in a group consisting of men rather than women (Derks et al., 2006). Finally, in terms of performance, women’s performance has been found to be negatively affected by activation of negative gender stereotypes (Cadinu et al., 2006), in groups where women are underrepresented compared to a group with equal gender composition (Inzlicht and Ben-Zeev, 2000), and by brief interactions with a sexist male confederate (Logel et al., 2009). This work demonstrates that gender identity threat is a situational predicament, evoked in response to the activation or salience of gender inequality or bias (see also Derks et al., 2006, 2008, 2016).

In comparison to the bulk of lab research on short-lived effects of contextual cues and primes on gender identity threat, the knowledge base in relation to prolonged exposure to male dominated work contexts in naturalistic settings is relatively small (Kalokerinos et al., 2014; Kang and Inzlicht, 2014). In naturalistic work settings, personal factors such as high competence, strong motivation, and positive past experiences may override classic context effects of social identity threat (Sackett, 2003; Sackett and Ryan, 2012). Given that women who opt for a career in STEM have clearly proven their competence, motivation and perseverance in STEM, one could argue that they have developed strategies to effectively cope with gender identity threats, or that they are resilient to them altogether.

However, recent field studies suggest that women working in male dominated work contexts do experience gender identity threats. For example, in the law and consumer industry, the more women compared themselves with their male (but not female) colleagues, the higher they scored on gender identity threat and the lower their career aspirations (Von Hippel et al., 2011). In the police force, the more women experienced gender bias, the higher their self-reported gender identity threat (Derks et al., 2011b) and the lower their perceived fit and belonging at work (Peters et al., 2013; Veldman et al., 2017). In the STEM sector, recent diary studies showed that women (but not men) engineers’ conversations with their male (but not female) colleagues cued feelings of incompetence and lack of acceptance. Moreover, on days that these conversations took place, levels of self-reported gender identity threat were higher (Hall et al., 2015, 2018a,b). Building on this work, in the current study we move from a micro level focus on daily interactions or cues that trigger gender identity threats at work, to a macro level focus on institutional parameters of male dominance that may cause gender identity threats among women STEM graduates working in- or outside the STEM sector.

### Numerical and Normative Male Dominance Elicit Gender Identity Threat Among Women in STEM

In professional fields such as the armed forces, the financial sector, academia, or the high-tech industry, male-dominance does not take a single form, but is often institutionalized in multiple ways. It is likely a *combination* of contextual parameters such as gender composition, gender stereotypes or biases that may elicit gender identity threats among women in these professional fields. Thus far, research often either did not clearly formulate the source of threat in response to male-dominance at work (Logel et al., 2009; Hall et al., 2015, 2018a), or focused on one such parameter at a time (e.g., Inzlicht and Ben-Zeev, 2000; Cadinu et al., 2006; Alt et al., 2017). What triggers threat responses among women in male dominated work contexts in STEM? The fact that women work in a sector in which few other women are present, the fact that they work in a sector that is stereotypically more strongly associated with masculine than feminine attributes, or a combination?

In conceptualizing institutional parameters that signal male dominance at work, we build on a sociological theory called the *double dominance theory* (Gruber and Morgan, 2005). This theory posits that institutional parameters of male dominance can be distinguished in two categories, namely (1) *numerical* and (2) *normative* male dominance. Numerical male dominance indicates the ratio of men to women in a work environment. The higher numerical male dominance is, the lower the proportion of women in an institution is. In this research, numerical male dominance is studied based on STEM graduates’ estimate of the ratio of men to women in their direct work environment. With only 13% of all STEM professionals being female, numerical male dominance in the STEM sector in the Netherlands is generally high, also relative to other sectors (Statistics Netherlands, 2016).

Normative male dominance indicates the extent to which a professional culture positively evaluates stereotypically masculine attributes (e.g., individualism, status-orientated) and/or negatively stereotypes women or feminine attributes (e.g., women are negatively stereotyped as incompetent in math). In this research, normative male dominance is studied by comparing professionals working either in- or outside the STEM sector. The STEM sector is stereotypically considered masculine (Diekman et al., 2010) and portrayed as highly competitive, individualistic, task-focused, high in status and monetary reward, and only carved out for those who are “brilliant” or “innately talented” (Leslie et al., 2015; Storage et al., 2016). These characteristics are typically attributed more to men more than to women. A recent cross-national survey among 66 countries revealed that people implicitly associate STEM abilities more strongly with men relative to women and the overall magnitude of this effect is large (Miller et al., 2015; see also Nosek et al., 2009). The Netherlands is a typical case in point, because despite the fact that the Dutch score relatively high on overall gender equity, Netherlands ranks first on explicit gender stereotypes, and second on implicit gender stereotypes in STEM (Miller et al., 2015).

In sum, based on double dominance theory (Gruber and Morgan, 2005), we posit that numerical and normative male dominance also have explanatory power in social identity research, and specifically on women’s experience of gender identity threat at work. We rely on recent field research demonstrating that women but not men report higher levels of gender identity threat in response to contextual cues signaling male-dominance (Hall et al., 2015, 2018a), to argue that high numerical and normative male-dominance at work also elicit high gender identity threat among women but not men STEM graduates. Moreover, we explore whether the combination of numerical and normative male dominance results in an interaction-effect, such that both reinforce each other to instill the highest levels of gender identity threat at work:

Hypothesis 1: The stronger both numerical and normative male dominance are at work, the higher gender identity threat among women but not men STEM graduates will be.

#### The Moderating Effect of Gender Identification

Importantly, not all women deal with threats to their gender identity in a similar manner (Ellemers et al., 2002; Schmader, 2002). The extent to which women in STEM may feel threatened in male-dominated work environments is expected to depend on their level of gender identification. Following from SIT (Tajfel and Turner, 1979), the more importance or self-relevance women attach to their gender identity (i.e., high gender identification), the more motivated they will be to maintain or protect a positive image of that gender identity (Tajfel and Turner, 1986; Ellemers et al., 1999) and hence, the greater the experience of gender identity threat in a context that signals male-dominance (Schmader, 2002; Major et al., 2003).

Building on this, we expect that when gender identity is considered highly self-relevant, confrontation with numerical or normative male dominance at work is more threatening for women professionals. In contrast, when gender identity is not considered very self-relevant, such dissociation from one’s gender identity can act as a coping mechanism to buffer against the negative feeling of being devalued or stigmatized at work on the basis of being a woman. Prior research on the Queen Bee phenomenon indeed demonstrates that women who report low connection to their gender group at work tend to distance themselves from this group to ward off potential gender identity threats and to successfully fit into a masculine work context (Derks et al., 2011a,b; see Derks et al., 2016 for review). Men’s gender identity may also play a role in their career-related perceptions, but rather in the sense that STEM careers are typically positively associated with masculine attributes (e.g., Diekman et al., 2010; Leslie et al., 2015). Thus, considering that men’s gender identity is generally not stigmatized in male dominated STEM work contexts we do not expect gender identification to act as a moderating mechanism among male STEM professionals:

Hypothesis 2: The effect of numerical and normative male dominance on women’s but not men’s, gender identity threat is moderated by gender identification, such that it is stronger among high relative to low identified women.

### Gender Identity Threat Negatively Affects Career Perceptions of Women in STEM

Social identity threats have negative consequences, such as for overall levels of cognitive functioning, decision-making, self-regulation, well-being, belonging, and self-esteem (e.g., Davies et al., 2002; Walton and Cohen, 2007; Inzlicht and Kang, 2010; Thoman et al., 2013). Following from this, we argue that women’s experience of gender identity threat in response to a male dominated work context in STEM negatively affects their career perceptions, particularly impairing work engagement and career confidence.

Work engagement can be defined as a positive, fulfilling work-related state of mind, characterized by high levels of energy, mental resilience, high involvement, and enthusiasm in one’s work (Schaufeli et al., 2002). The more work engagement people experience, the higher their commitment to their organization (Hakanen et al., 2008), and the lower their intentions to leave (Du Plooy and Roodt, 2010). Past research focussed on job-level (e.g., job autonomy, learning opportunities) and individual-level (e.g., self-esteem, optimism) processes as main driving forces of work engagement (Bakker and Demerouti, 2008), while little attention has been devoted to group-level processes. We argue that when women STEM professionals have to deal with gender identity threats in response to a male dominant work context, this requires cognitive and emotional resources that take away from their enthusiasm and involvement in their work. In empirical support for this, research showed that feeling negatively stereotyped as a female STEM student contributed to higher disengagement and lower interest to continue a career in STEM (Davies et al., 2002; Cheryan et al., 2009; Thoman and Sansone, 2016). Moreover, diary studies showed that on days that female STEM faculty engaged in research conversations with male colleagues, their reported disengagement at work was higher, while the reverse was true for male STEM faculty (Holleran et al., 2011). Moreover, on days that female, but not male, engineers interacted more with their male colleagues, they experienced more gender identity threat and as a consequence, reported higher levels of burn-out (i.e., being emotionally drained and exhausted at work; Hall et al., 2015, 2018a).

Hypothesis 3: Higher levels of gender identity threat in response to male dominated work contexts are associated with lower levels of work engagement among, female, but not male, STEM professionals.

Career confidence can be defined as the overall certainty or clarity that people experience about their future career perspectives. People with high career confidence know what they want in their career and are confident that they will be able to achieve their career goals (Savickas and Porfeli, 2011; Gupta et al., 2015). Research based on social cognitive career theory (SCCT; Lent et al., 1994) showed that female college students’ confidence in their own ability to perform well in a STEM study, positively affected their interest and choice for a career in the STEM sector (Lent et al., 2003, 2005; Cech et al., 2011). Moreover, female engineers’ positive beliefs in their competence in STEM positively predicted their commitment and negatively predicted their turnover intentions in STEM (Singh et al., 2013). Finally, compared to female engineering graduates who previously worked in engineering but left, those who still worked in engineering report higher levels of domain-specific STEM confidence (Fouad et al., 2016). Integrating this work on socio-cognitive career theory with theory on social identity processes at work, we argue that gender identity threat forms an important explanatory mechanism as to why male dominated work contexts impose a contextual barrier for female STEM graduates’ career confidence. Initial support among student samples showed that female STEM students’ experience of gender identity threat in male-dominated educational contexts lowered their self-efficacy (Deemer et al., 2014) and career motivation (see for review Thoman et al., 2013) in STEM. Thus, we hypothesize that:

Hypothesis 4: Higher levels of gender identity threat in response to male dominated work contexts, are associated with lower levels of career confidence among, female, but not male, STEM professionals.

## Materials and Methods

### Participants and Design

In a cross-sectional field study^1^ performed in the Netherlands, 877 STEM graduates filled out an online survey. Forty-five participants dropped out at an early stage and were excluded from further analyses (this drop out was random across men and women χ*^2^*(1) = 0.34, *p* = 0.56). Twelve participants had missing values on the covariates in the model (age, contract size and educational level) and were excluded from analyses. Because we only focused on STEM graduates with paid work, or who had had paid work within the past 12 months, another 13 participants were excluded. In total, 807 participants were included for analysis. Of these participants, 630 were men (78%) and 177 were women (22%)^2^. In terms of educational level, 69% completed a scientific educational STEM program at a University, and 31% completed a higher vocational educational STEM program at a University of Applied Sciences. The average contract size (in hours per week) was 36.62 (*SD* = 7.4). For women, the average contract size was 35.45 (*SD* = 7.14) hours per week, and for men the average contract size was 36.96, (*SD* = 7.48) hours per week*, t*(805) = 2.40, *p* = 0.02, *95% CI:* 0.27_lb__-_ 2.75_*ub*._ The average age of participants was *M* = 35.77 (*SD* = 10.74).

### Instruments and Procedure

An online survey was distributed among all graduates from STEM study programs, via the alumni offices of two educational institutions in the Netherlands. Permission was asked to contact the alumni offices via the educational directors of all STEM educational programs. The study was approved by the Ethics Committee of the Behavioural Science and Management Faculty at the University of Twente. STEM graduates were contacted via their alumni email addresses. In total, 24,402 STEM alumni from the University and 6,035 STEM alumni from the Higher Vocational Education Institute were contacted and invited to participate in the research. From the alumni who graduated at University, 560 responses were analyzed (response rate: 2.3%) and from alumni who graduated at the Higher Vocational Education institute, 247 responses were analyzed (4.1%). Overall response rates were low and this is likely due to the fact that alumni email addresses are generally not used actively by graduates; we suspect the vast majority did not read the invitation email.

In the invitation email, STEM alumni were informed that the purpose of the study was to gain insight in the career choices that STEM graduates make after they finish their education in order to better prepare current STEM students in their labor market perspectives. A web link was provided in the email that redirected participants to the questionnaire. Online informed consent was obtained from all participants. After the general introduction, participants were asked questions about their demographic and professional background, their current work situation, their career perceptions and about the role of their gender identity at work. Unless reported otherwise, items were based on a 7-point Likert scale (1 = I totally disagree; 7 = I totally agree). It took participants about 20 min to finish the survey.

#### Normative and Numerical Male Dominance

To measure normative male dominance, we asked participants to indicate whether they currently worked either in the STEM sector or in a non-STEM sector. In total, 77% indicated to work in the STEM sector. Among women, this percentage was 63% and among men, it was 81%, χ*^2^*(1) = 27.61, *p* < 0.001. Specifically, of all female participants, 111 worked in the STEM sector and 66 worked outside STEM. Of all male participants, 513 worked in STEM sector and 117 worked outside STEM. While the groups differ in size, within both gender groups sample sizes are such that they do allow for making reliable statistical inferences (Van Voorhis and Morgan, 2007).

Secondly, to measure numerical male dominance at work, we asked participants to estimate the ratio of women relative to men in their direct work environment (i.e., gender ratio). Participants could answer on a 5-point scale (1 = no women, only men; 2 = some women, mostly men; 3 = an equal amount of women and men; 4 = mostly women, no men; 5 = only women no men). Thus, higher scores indicated a *higher* ratio of women relative to men in the direct work environment (and thus *lower* numerical male dominance). Men indicated a stronger underrepresentation of women in their direct work environment (*M* = 2.16, *SD* = 0.56) relative to women (*M* = 2.65, *SD* = 0.82), *t*(224.83) = -7.41, *p* < 0.001, *CI_95%_* : -0.62 - -0.38 ^3^. Importantly, both genders indicated, on average, that the gender distribution was skewed such that men outnumbered women at work.

#### Gender Identification at Work

Gender identification at work was measured with four items taken from Derks et al. (2011a). The items were, “At work, I feel closely connected to other people of my own gender,” “At work, I identify with people of my own gender,” “At work, I feel committed to people of my own gender,” and “At work, being a woman/man is important to me” (α = 0.80).

#### Perceived Gender Identity Threat

To measure perceived gender identity threat at work we adopted four items from Hall et al. (2015). We introduced the questions by stating: *“Think about the day-to-day work activities and interactions that you have in your work. To what extent do you agree with the following statements?”* The items were: “I am often aware of the fact that I am a woman/man when I interact with others at work,” “Sometimes I am concerned that being a woman/man influences how others see me professionally,” “It worries me sometimes that others might judge my work on the basis of my gender,” and “Sometimes I feel uncomfortable at work because I am a woman/man” (α = 0.84).

#### Career Confidence

Career confidence was measured with six items adapted from Savickas and Porfeli (2011) and Gupta et al. (2015) career adaptability scales. Items were: “I know what I want in my career,” “I have a clear sense of what I want to achieve in my career,” “I have confidence in my career,” “I keep changing my mind about what I want in my career’ (reverse scored), “I often think that I should change things in my career” (reverse scored), and “I am uncertain about the choices I want to make in my career” (reverse scored; α = 0.83).

#### Work Engagement

Work engagement was measured with two items from Schaufeli et al. (2006), namely “At work I feel strong and vigorous” and “When I get up in the morning, I feel like going to work,” *r*(807) = 0.67, *p* < 0.001.

## Results

### Descriptive Statistics

In Table 1, means (*M)* and standard deviations (*SD*) on model variables are displayed, separately for men and women. In addition, *t*-tests were included to test for gender differences, correcting for a potential violation of equal variances across gender groups (men are overrepresented in the dataset relative to women). Compared to men, women reported to work in STEM less often and reported lower numerical male dominance (i.e., gender ratio) in their work context. Moreover, women reported higher gender identification, higher gender identity threat and lower career confidence compared to men. No significant gender differences were found on work engagement.

**Table 1:** Descriptive statistics on model variables of total sample (*N* = 807), women (*N* = 177), and men (*N* = 630) separately, and *t*-tests and 95% CI on gender differences.

		*M*	*SD*				*95% CI*	
				*t*	*df*	*p*	*lb*	*ub*
Work sector^∗^_(0__=__STEM;1__=__non-STEM)_	Women	0.37	0.49					
	Men	0.19	0.39	4.73	243.23	<0.001	0.11	0.27
	Total	0.23	0.42					
Gender ratio at work^∗^	Women	2.65	0.82					
	Men	2.16	0.56	7.41	224.83	<0.001	0.36	0.62
	Total	2.27	0.66					
Gender identification	Women	3.74	1.15					
	Men	3.44	1.22	2.91	805	0.004	0.10	0.50
	Total	3.51	1.21					
Gender identity threat^∗^	Women	3.12	1.45					
	Men	1.85	0.88	11.09	214.11	<0.001	1.04	1.49
	Total	2.13	1.16					
Career confidence	Women	4.46	1.24					
	Men	4.77	1.12	–3.02	805	0.001	–0.51	–0.12
	Total	4.70	1.16					
Work engagement	Women	5.00	1.22					
	Men	5.12	1.16	–1.24	805	0.216	–0.32	0.07
	Total	5.10	1.18					


*^∗^t-test results corrected for equal variances not assumed across gender groups where provided as Levene’s test was significant for these variables. There were no differences in *t*-test results between corrected and uncorrected tests. A higher score on gender ratio indicates a higher representation of women in the direct work environment.*

In Table 2, correlations between variables in the model are displayed, separately for men and women. Only women but not men, reported higher gender identity threat when they worked in the STEM sector compared to non-STEM sectors. Only among women but not men, the higher the reported ratio of women in the work context relative to men, the lower the levels of reported gender identity threat. Gender identity threat at work was negatively related to career confidence among both men and women, and negatively related to work engagement, only among women. There was a positive correlation between normative (STEM vs. non-STEM) and numerical (gender ratio) male dominance; this reflects the situation in the Netherlands that the STEM sector is, numerically speaking, the most male dominated sector relative to other economic sectors (Statistics Netherlands, 2016).

**Table 2:** Correlations between model variables separately for gender groups.

	1.	2.	3.	4.	5.	6.
1. Work sector _(0__=__STEM;1__=__non-STEM)_	–	0.530***	0.009	–0.272***	–0.019	0.058
2. Gender ratio	0.347***	–	–0.022	–0.436***	0.032	0.054
3. Gender identification	–0.120**	–0.107**	–	0.273***	0.045	0.053
4. Gender identity threat	–0.023	0.000	0.267***	–	–0.212**	–0.151*
5. Career confidence	–0.071	–0.039	0.032	–0.098**	–	0.434***
6. Work engagement	–0.009	0.085*	0.100*	–0.030	0.475***	–


*Women (*N* = 177) are displayed above the diagonal; men (*N* = 630) are displayed below the diagonal. ^∗∗∗^Correlation is significant at the 0.001 level. ^∗∗^Correlation is significant at the 0.01 level. ^∗^Correlation is significant at the 0.05 level. A higher score on gender ratio indicates a higher representation of women in the direct work environment.*

To gain more insight in gender differences in reported gender ratio in the non-STEM sector, an ANOVA was conducted with gender and work sector (STEM vs. non-STEM) as independent variables and gender ratio as dependent variable. Results showed an interaction effect of Gender × Work Sector on gender ratio, *F*(1,803) = 13.80, *p* < 0.001, $\eta_{p}^{2}$ = 0.02. In the non-STEM sector, on average women indicated to work in a context with an equal gender distribution (*M* = 3.21, *SD* = 0.80), while men still reported to work in a context with a majority of men (*M* = 2.57, *SD* = 0.70), *F*(1,803) = 53.07*, p* < 0.001, $\eta_{p}^{2}$ = 0.06. In the STEM sector, both women (*M* = 2.32, *SD* = 0.62) and men (*M* = 2.07, *SD* = 0.48) reported to work in a male dominated work context, yet men reported this gender ratio to be significantly more skewed (i.e., more male dominance) than women, *F*(1,803) = 16.87*, p* < 0.001, $\eta_{p}^{2}$ = 0.02. In addition, the variables measuring numerical and normative male dominance were correlated (among women: *r* = 0.53; among men *r* = 0.35), but this level of multicollinearity is still considered small to moderate and therefore unlikely to result in Type II error, also given our relatively large sample size [see Grewal et al., 2004 for more information on multicollinearity in Structural Equation Modeling (SEM; Muthén and Muthén, 1998/2012)]. Note that the independent variables will covary in the SEM model, enabling us to draw inferences about the unique variance explained by both parameters of male dominance (i.e., numerical and normative) on gender identity threat and career perceptions.

### Analytical Strategy

The conceptual model (Figure 1) was tested with SEM using MPlus 8, to obtain maximum likelihood estimates (ML) with robust standard errors and a robust chi-square measure of overall goodness of fit. The fit of a SEM model is considered good when the root mean squared error of approximation (RMSEA) and the standardized root mean square residual (SRMSR) are ≤0.06 and the comparative fit index (CFI) and the Tucker–Lewis Index (TLI) are ≥0.90. Finally, the χ^2^> 0.05 and the value of χ^2^, divided by the degrees of freedom should be less than 3 (Hu and Bentler, 1999; Kline, 2015).

To investigate whether the hypothesized structural equation model would differ between men and women, we applied multi-group analyses and compared model fit indices when parameter estimates are constraint (expected to be similar) or freed (expected to be different) across gender groups (Geiser, 2012). To investigate whether normative (i.e., working in the STEM sector) and numerical (gender ratio) male dominance in the work context would impact on female STEM professionals’ gender identity threat, whether both variables would interact (Hypothesis 1) and whether they would be moderated by gender identification (Hypothesis 2) we Z-standardized continuous variables and computed the two-way interaction terms (Aiken et al., 1991) and estimated parameter estimates on gender identity threat. Moreover, we estimated parameter estimates from gender identity threat to work engagement and career confidence. To test the proposed mediation of gender identity threat between (male dominated) work context and career perceptions (Hypotheses 3 and 4) we performed indirect effects testing by generating bootstrapped confidence intervals (5,000 iterations; Shrout and Bolger, 2002; MacKinnon et al., 2004).

### Model Fit

We compared the hypothesized model against a baseline model (null-model) to test overall fit to the data. In the baseline model, none of the paths between variables are expected to be significant. This model obtained bad model fit, χ^2^(60) = 441.34, *p* < 0.001, χ^2^/df = 7.36. In the hypothesized model, we added Z-standardized regression paths from gender identification, gender ratio, work sector (0 = STEM; 1 = non-STEM) and their two-way interaction terms to gender identity threat^4^. Moreover, we added regression paths from gender identity threat to career confidence and work engagement. Correlational paths were added between career confidence and work engagement and all independent variables were allowed to covary. Age, contract size (hours per week) and educational level (0 = University of Applied Sciences; 1 = University) served as covariates. The hypothesized model obtained good fit (χ^2^[30] = 48.95, *p* = 0.016, χ^2^/df = 1.63, RMSEA = 0.040, SRMR = 0.022. CFI = 0.95, TLI = 0.90) and was significantly better compared to the baseline model, Δχ^2^ (60) = 392.39 *p* < 0.001. Overall, we concluded that our hypothesized model was a good fit to the data.

### Hypothesis Testing

**FIGURE 2:**
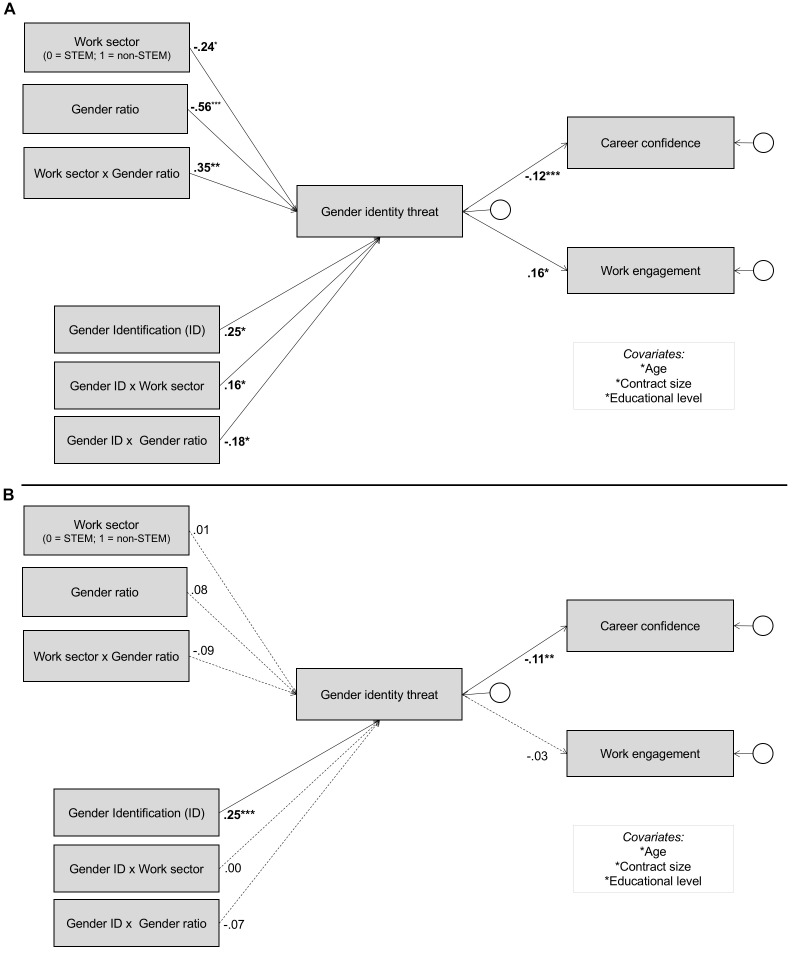
**(A)** Structural Equation Model for women (*N* = 177). Significant standardized parameter estimates marked in bold. **(B)** Structural Equation Model for men (*N* = 630). Significant standardized parameter estimates marked in bold; non-significant standardized parameter estimates are indicated with a dotted line.

In order to test whether the proposed relationships in our model would be different for women compared to men, we conducted multi-group comparisons. Here, the model fit of the unconstrained, hypothesized model (paths were allowed to vary between men and women) was compared to the constrained model (paths were not allowed to vary), χ^2^(45) = 101.65, *p* < 0.001, χ^2^/df = 2.26. The difference between models was significant, Δχ^2^(15) = 52.70, *p* < 0.001 indicating that the relationship between male dominant work contexts (normative and numerical), gender identification, gender identity threat, and career perceptions were different between men and women. We conducted path-by-path comparisons based on Δχ^2^ testing on the parameter estimates between men and women to investigate where moderation occurs (see Table 3). We discuss the parameter estimates in relation to our hypotheses. See Figure 2A,B for standardized parameter estimates in the SEM model for men and women.

**Table 3:** Standardized direct and indirect effects parameter estimates and path-by-path analysis on Δχ^2^ for both gender groups (women *N* = 177; men *N* = 630) separately.

			Women		Men		
			Estimate	*p*	Estimate	*p*	Δχ^2^
**Independent variables → gender identity threat**							
Work sector _(0__=__STEM;1__=__non-STEM)_	→	Gender identity threat	–0.24	0.012	0.01	0.875	6.00*
Gender ratio	→	Gender identity threat	–0.56	<0.001	0.08	0.319	29.23***
Gender ID	→	Gender identity threat	0.25	0.012	0.25	<0.001	1.57
Sector × ratio	→	Gender identity threat	0.35	0.005	–0.09	0.340	10.63**
Ratio × gender ID	→	Gender identity threat	–0.18	0.016	–0.07	0.228	1.70
Sector × gender ID	→	Gender identity threat	0.16	0.048	0.00	0.981	2.64
**Gender identity threat → career perceptions**							
Gender identity threat	→	Career confidence	–0.12	0.001	–0.11	0.007	0.04
		Work engagement	–0.16	0.034	–0.03	0.348	1.22
**Covariates**							
Age	→	Career confidence	0.19	0.022	0.26	<0.001	0.13
		Work engagement	0.18	0.041	0.18	<0.001	0.47
Contract size	→	Career confidence	0.14	0.053	0.03	0.499	1.95
		Work engagement	0.16	0.019	0.13	0.003	0.21
Education level _(0=applieduniversity;1=university)_	→	Career confidence	0.25	0.004	0.12	0.018	1.88
		Work engagement	0.18	0.036	0.04	0.463	1.94


*^∗∗∗^Δχ^*2*^ is significant at the 0.001 level. ^∗∗^Δχ^*2*^ is significant at the 0.01 level. ^∗^Δχ^*2*^ is significant at the 0.05 level. A higher score on gender ratio indicates a higher representation of women in the direct work environment.*

#### Hypothesis 1: Numerical and Normative Male Dominance Elicit Gender Identity Threat Among Women STEM Professionals

**FIGURE 3:**
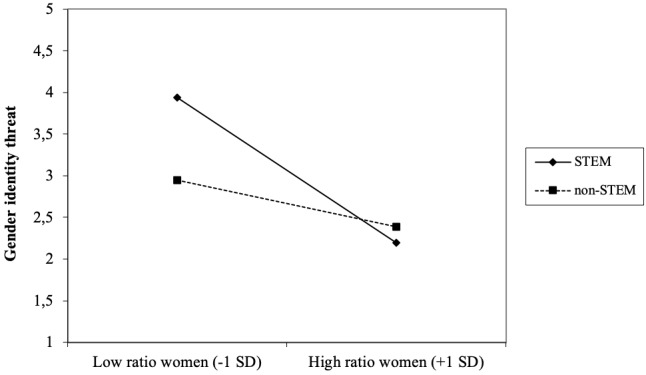
Two-way interaction-effect gender ratio × work sector (STEM vs. non-STEM) on gender identity threat among women.

In support of Hypothesis 1, specifically among women but not men, those who indicated to work in the STEM sector reported higher levels of gender identity threat at work than those who did not work in the STEM sector (γ = -0.24, *SE* = 0.09, *p* = 0.010). Moreover, the higher the ratio of women relative to men in the work context, the lower women’s but not men’s reported levels of gender identity threat (γ = -0.56, *SE* = 0.90, *p* < 0.001). Moreover, specifically women but not men working in STEM faced a double identity threat in male dominated work contexts; the interaction effect between work sector (STEM vs. non-STEM) and gender ratio among women was significant (γ = 0.35, *SE* = 0.12, *p* = 0.005; see Figure 3). Simple slope analysis revealed that women who worked in the STEM sector (normative male dominance) *and* reported a highly skewed male-to-female ratio in their work context (numerical male dominance) experienced highest levels of gender identity threat. Specifically, for women working in the STEM sector, gender identity threat increased significantly as the ratio of women to men decreased, *b* = -0.71, *t*(176) = -5.27, *p* < 0.001. While a similar trend was found for women working in non-STEM sectors, the relationship between gender ratio and gender identity threat was not significant, *b* = -0.23, *t*(176) = -1.95, *p* = 0.052. Put differently, when the ratio of women to men was reported as relatively high (*M*+1 SD), there was no evidence that work sector (STEM vs. non-STEM) affected experienced gender identity threat, *b* = -0.23, *t*(176) = -0.98, *p* = 0.33. However, when the ratio of women to men in the work context was reported as low (*M*-1 SD; e.g., strong underrepresentation of women), women working in the STEM sector reported significantly higher levels of gender identity threat relative to women outside STEM, *b* = -0.71, *t*(176) = -2.47, *p* = 0.015. Thus, numerical underrepresentation of women in the work context forms a source of gender identity threat, more so for women working in- than outside the STEM sector.

#### Hypothesis 2: Effects of Male Dominance at Work Are Stronger for Women With High Gender Identification

Gender identification was significantly associated with experienced gender identity threat, such that the higher individuals identified with their gender identity at work, the higher the gender identity threat they experienced at work. This was the case for both men (γ = 0.25, *SE* = 0.05, *p* < 0.001) and women (γ = 0.25, *SE* = 0.08, *p* = 0.001). Importantly, in support for Hypothesis 2, specifically for women, the effect of gender identification on gender identity threat was contingent upon both numerical and normative male dominance at work; both the two-way interaction-effect between work sector (STEM vs. non-STEM) and gender identification (γ = 0.16, *SE* = 0.08, *p* = 0.048), as well as the interaction effect between gender ratio and gender identification was significant (γ = -0.18, *SE* = 0.07, *p* = 0.012), for women but not men^5^.

**FIGURE 4:**
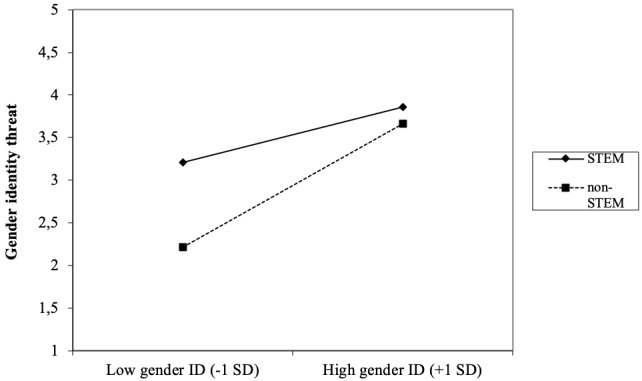
Two-way interaction-effect work sector (STEM vs. non-STEM) × gender identification on gender identity threat among women.

In Figure 4 the interaction-effect between work sector and gender identification is displayed. Simple slope analysis revealed that for women in non-STEM sectors, gender identity threat was significantly higher among high compared to low identifiers, *b* = 0.73, *t*(176) = 4.09, *p* < 0.001. Similar but weaker results for gender identification were found among women in STEM, *b* = 0.33, *t*(176) = 2.48, *p* = 0.014. Moreover, women who strongly identified with their gender identity (*M*+1 *SD*) reported similarly high levels of gender identity threat at work, irrespective of whether they worked in- or outside STEM, *b* = -0.20, *t*(176) = -0.62, *p* = 0.54. Women who identified less strongly with their gender identity (*M -*1 *SD*) reported significantly higher levels of gender identity threat when working in STEM relative to working in a non-STEM sector, *b* = -1.03, *t*(176) = -2,73 *p* = 0.007.

**FIGURE 5:**
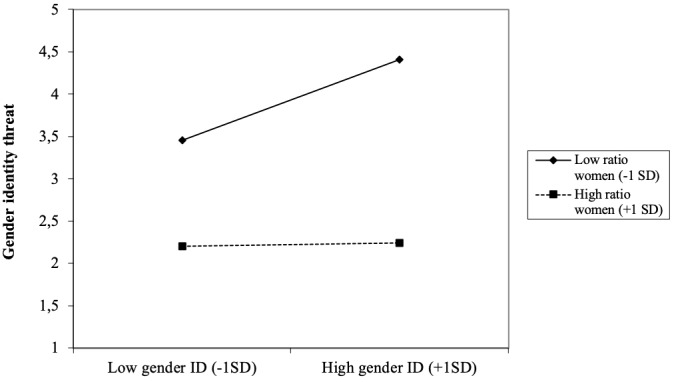
Two-way interaction-effect gender ratio × gender identification on gender identity threat among women.

In Figure 5, the interaction effect between gender ratio at work and gender identification is displayed. Simple slope analysis revealed that when women were strongly underrepresented relative to men at work (*M*-1 *SD*; low ratio women), gender identity threat was significantly higher among high compared to low identifiers, *b* = 0.50, *t*(176) = 3.79, *p* < 0.001. When women and men were approximately equally represented at work (*M*+1 *SD*; high ratio women, *M* = 2.93 on 5-point scale, with 3 indicating equal gender representation), gender identity threat was relatively low, and there was no evidence for an association with gender identification, *b* = 0.02, *t*(176) = 0.12, *p* = 0.91. Moreover, women who worked in a context with an approximately equal gender distribution experienced lower levels of gender identity threat relative to those who worked in a male dominated context; this was the case for both low (*M -*1 *SD*; *b* = -0.51, *t*(176) = -3.71, *p* < 0.001) and high [*M* +1 *SD*; *b* = -0.87, *t*(176) = 5.54, *p* < 0.001] gender identifiers.

#### Hypotheses 3 and 4: For Women, Gender Identity Threat Mediates the Relationship Between Male Dominance and Career Perceptions

We hypothesized that to the extent that normative and numerical male dominance at work form a source of gender identity threat among women STEM graduates, this would have negative consequences for their career outcomes, namely work engagement (*Hypothesis 3*) and career confidence (*Hypothesis 4*). To test these indirect effects, we generated 95% bias-corrected bootstrapped confidence intervals (CI) on indirect effects (5,000 iterations; Shrout and Bolger, 2002; MacKinnon et al., 2004). Moreover, we imposed model constraints on the indirect effects with Δχ^2^ testing to investigate whether indirect effects were different across gender groups (Ryu and Cheong, 2017).

First, with respect to work engagement (Figure 2 and Table 3), results showed that gender identity threat was significantly negatively related to work engagement among women (γ = -0.16, *SE* = 0.07, *p* = 0.034), but no evidence was found for such relationship among men (γ = -0.03, *SE* = 0.04, *p* = 0.384). Note however that the Δχ^2^ test of the direct effect between gender identity threat and work engagement across gender groups was not significant. In Table 4, *CI_95%_* for the indirect effects in the SEM model are displayed. Results showed a significant indirect effect of work sector (STEM vs. non-STEM), gender ratio, and the interaction term between work sector and gender ratio on work engagement via gender identity threat among women, while no such evidence was found among men. This difference was significant between gender groups. That is, in line with Hypothesis 3, to the extent that normative and numerical male dominance form a source of gender identity threat among women, this negatively affected their work engagement.

**Table 4:** Indirect effects testing with 95% bias-corrected bootstrapped confidence intervals (CI) on the mediating effect of gender identity threat (M) between independent variables (X) and work outcomes (Y), for men and women separately.

			Women			Men			
			Indirect effect	*CI_95%_*		Indirect effect	*CI_95%_*		Δχ^2^
				*LB*	*UB*		*LB*	*UB*	
**Indirect effect X → Y via gender identity threat (M)**									
Work sector _(0=STEM;1=non-STEM)_	→	Work engagement	0.036	0.003	0.087	0.000	–0.008	0.004	6.41**
		Career confidence	0.051	0.009	0.109	–0.001	–0.014	0.010	6.21**
Gender ratio	→	Work engagement	0.120	0.046	0.213	–0.008	–0.025	0.011	10.13***
		Career confidence	0.087	0.007	0.175	–0.002	–0.016	0.006	4.80*
Gender ID	→	Work engagement	–0.053	–0.119	–0.012	–0.026	–0.051	–0.010	1.75
		Career confidence	–0.038	–0.102	–0.003	–0.008	–0.025	0.009	2.26
Sector × ratio	→	Work engagement	–0.075	–0.163	–0.019	0.010	–0.022	0.024	10.04**
		Career confidence	–0.054	–0.135	–0.007	0.003	–0.013	0.018	8.18**
Ratio × gender ID	→	Work engagement	0.039	0.006	0.088	0.008	–0.005	0.024	2.11
		Career confidence	0.028	0.001	0.077	0.002	–0.004	0.015	2.87
Sector × gender ID	→	Work engagement	–0.035	–0.092	–0.001	0.000	–0.022	0.012	2.83
		Career confidence	–0.026	–0.077	0.000	0.000	–0.007	0.005	3.07


*^∗^*p* ≤ 0.05; ^∗∗^*p* ≤ 0.01; ^∗∗∗^*p* ≤ 0.001.*

Second, with respect to career confidence results from the parameter estimates (Figure 2 and Table 3) showed that gender identity threat was significantly negatively related to career confidence among both women (γ = -0.22, *SE* = 0.07, *p* = 0.001) and men (γ = -0.11, *SE* = 0.04, *p* = 0.007). In Table 4, *CI_95%_* for the indirect effects in the SEM model are displayed. Results showed a significant indirect effect of work sector (STEM vs. non-STEM), gender ratio, and the interaction term between work sector and gender ratio on career confidence via gender identity threat among women, while no such evidence was found among men. This gender difference was significant for both the main-effects and the interaction-effects. That is, in line with Hypothesis 4, to the extent that normative and numerical male dominance form a source of gender identity threat among women, this negatively affected their career confidence.

Third, results in Table 4 also showed that for both women and men, gender identification indirectly predicted their career confidence via gender identity threat; the more STEM graduates identified with their gender identity at work, the more gender identity threat they experienced, with lower career confidence as a down-stream effect. For women, we found that this indirect effect of gender identification was also contingent upon the gender ratio (i.e., numerical dominance) in the direct work environment. That is, particularly women who were highly identified with their gender identity and who were also strongly outnumbered by men in their work context were negatively affected in their career confidence via high levels of gender identity threat. Importantly, however, while the indirect effect of the interaction term between gender identification and gender ratio was significant among women but not men, the Δχ^2^ of this indirect effect was not significant.

Finally, it is interesting to note that the variance explained by numerical (gender ratio) and normative (work sector) male dominance at work, gender identification and their two-way interaction terms on gender identity threat among women was *R*^2^ = 0.30, which boils down to an effect size of *f^2^* = 0.43 (large effect; Cohen, 1988). For men, the explained variance was *R*^2^ = 0.08, which boils down to an effect size of *f^2^* = 0.09 (small effect), driven only by gender identification. The explained variance for career confidence and work engagement was considerably smaller among both women (career confidence: *R*^2^ = 0.10; work engagement *R*^2^ = 0.07) and men (career confidence: *R*^2^ = 0.06; work engagement *R*^2^ = 0.04). Indeed, as prior research demonstrates, career confidence and work engagement also depend on individual- and organization-level factors.

## Discussion

The goal of this paper was to investigate how different institutional parameters of male dominance predict career perceptions of women in STEM. In doing so, we relied on double dominance theory (Gruber and Morgan, 2005) and distinguished between numerical and normative male dominance at work. We focussed on a unique population of professionals, namely highly educated female STEM graduates who opted for a career either in- or outside the STEM sector. We took a social identity lens (Tajfel and Turner, 1979) to put forward gender identity threat as an important mechanism to explain how masculine work contexts translate into career barriers for women in STEM.

### Numerical and Normative Male Dominance Have Unique and Combined Effects on Gender Identity Threat

Study results showed that the more women reported to be outnumbered by men in their direct work environment (i.e., *numerical* male dominance), the higher their experience of gender identity threat was. Following from the social identity approach (Tajfel and Turner, 1979), being one of the only few women at work means being highly dissimilar from most other colleagues. This makes one’s gender category highly salient (Wilder, 1984; Turner et al., 1987), increases awareness about one’s gender at work, and heightens the expectation that one will be viewed by others in terms of one’s gender category (Frey and Tropp, 2006). In line with prior research, our data revealed that numerical male dominance thus gives rise to gender identity threats among female STEM graduates (e.g., Murphy et al., 2007; Veldman et al., 2017).

Above and beyond women’s numerical male dominance, the mere fact of working in the STEM vs. non-STEM sector (i.e., *normative* male dominance) also uniquely predicted gender identity threat. Women working in STEM reported higher gender identity threat levels compared to women working in a non-STEM sector. Traditionally male dominant professional cultures such as STEM tend to be associated with a higher value attached to the male identity and to typically masculine characteristics than the female identity and typically feminine characteristics (Branscombe and Ellemers, 1998; Derks et al., 2006, 2018; Van Laar et al., 2010). Indirect support that this is the case in our data can be inferred from the fact that male professionals working in STEM identified more strongly with their gender identity at work than they did when working outside STEM (Table 2). Our results suggest that for women, working in the STEM sector elicits more gender identity threat than working outside the STEM sector – even among women who have successfully obtained an academic degree in STEM and have made the decision to continue their career in this field.

The *combination* of numerical and normative male dominance resulted in highest levels of gender identity threat among women STEM graduates. This is in line with what double-dominance theory would suggest (Gruber and Morgan, 2005). Thus far, this theory has been applied from a *perpetrator’s* perspective, to predict the prevalence of sexual harassment cases in work contexts where both numerical and normative male dominance are high (de Haas and Timmerman, 2010; Dresden et al., 2018). Expanding on this theory, in this study our primary focus was on the *target’s* perspective and the demonstration that for women, the joint experience of numerical and normative male dominance was associated with highest levels of gender identity threat. It could be speculated that the gender identity threat findings uncovered in this research are related to women’s actual experience of sexual harassment in male dominant work contexts (Leaper and Starr, 2018). Further combining these sociological and socio-psychological theories to investigate this connection might be an interesting avenue for future research.

Our investigation of social identity processes among a unique population of female and male STEM professionals contributes to recent research and theorizing on social identity threats in naturalistic work settings (Hall et al., 2015, 2018a,b). Also, it appeals to the growing call for research that seeks to understand social identity processes among women in STEM *after* they complete their education and enter the workplace (Walton et al., 2015). Adding to this knowledge base, our study demonstrates that social identity threats are not only evoked in response to temporary (e.g., daily) activation of situational cues that signal male dominance *within* STEM, but also that working in the STEM sector in itself (as opposed to outside STEM) serves as a source of gender identity threat among women professionals. This suggests that while women STEM graduates’ personal experience and ability in STEM may certainly contribute to their overall confidence and perseverance in STEM (Cech et al., 2011; Fouad et al., 2016), this does not completely override the fact that masculine STEM working contexts impose a threat on women’s gender identity and form barriers to their career advancement. Together, our findings enrich social identity research in organizations, extending its validity not only to short-lived, situational salience of gender inequality or bias at work, but also to prolonged exposure to biased institutional systems.

In terms of practical implications, our results point to the importance of the numerical representation of women for their work experiences, especially in the STEM sector. The reported gender ratio at work most strongly affected women’s experienced gender identity threat in our model, with negative consequences for their work engagement and career confidence. Moreover, this effect turned out to be even stronger for women working in STEM. This suggests that actions that increase the number of women working in STEM can have potent effects on women’s work experiences. The stronger the representation of women in STEM, the less gender identity threat women experience, and hence the stronger their work engagement and career confidence. This, in turn, may have important trickle-down effects that impact upon the masculine organizational culture within the STEM sector. For example, the more women feel confident and engaged at work and the less they worry about their gender identity, the more likely it is that they will be their authentic self, hereby adding to increased heterogeneity in perspectives in their company (Galinsky et al., 2015). Only when women add their perspectives rather than try to assimilate into masculine culture (e.g., Derks et al., 2016) will gender diversity actually lead to more optimal diverse human capital utilization.

### The Role of Gender Identification in Masculine Work-Contexts

The current results once again show that gender identification at work plays an important role in the extent to which masculine work contexts affect women’s experience of social identity threat. Specifically, our study showed that especially women who identified highly with their gender at work, were negatively affected by being strongly outnumbered by men in their work context. Put differently, when women were underrepresented at work, those who attributed the least significance to their gender identity were also the least affected by gender identity threats. This finding is in line with research showing that one identity strategy for women to protect themselves against gender identity threats in masculine work contexts is to *distance* the self from the gender identity at work (Ellemers et al., 2012; Derks et al., 2016; Faniko et al., 2017). Indeed, in a recent life history study, female associate and full professors in science tended to downplay or ignore the significance of gender when being interviewed about their career trajectory (Britton, 2017).

Gender identification also played a moderating role in relation to women’s gender identity threat depending on their work sector (STEM vs. non-STEM). While gender identity threat was generally higher when women worked in the STEM sector, especially in the non-STEM sector women’s experience of gender identity threat depended more strongly on their gender identification: in the non-STEM sectors, women’s low gender identification yielded lowest levels of gender identity threat. In line with recent work on ‘gender blindness’ (Martin and Phillips, 2017) this may suggest that when the relevance of women’s gender identity at work is low, both in the work context (non-STEM; low normative male dominance) and from the individual’s perspective (low gender identification) they are least likely to feel uncertain or uncomfortable at work on the basis of being a woman.

Importantly, however, this is not to say that we consider low gender identification at work an effective strategy to prevent women STEM professionals from experiencing identity threats. Firstly, while our results showed that lower gender identification was associated with lower reported gender identity threat, low identifiers were not completely immune to gender identity threat effects in male dominated work environments. The lowest identity threat levels were reported among women working either outside the STEM sector, or in an environment where gender representation was approximately equal. Secondly, low gender identification also has disadvantages, because it causes women to distance from other women, and to not support (or even oppose) collective actions directed at improving their low status position in masculine work contexts (e.g., Derks et al., 2016). As a consequence, low identified women in STEM also likely do not serve as a role model for the undergraduate female STEM students and their career decisions to stay or leave the STEM sector. Finally, high gender identification also has advantages. Following the rejection-identification model (Branscombe et al., 1999) gender identification can serve a protective function to cope with gender inequality, in that a sense of belongingness and acceptance in a minority group of women at work can provide a psychological buffer against hostile, male dominant work climates which lowers psychological distress (Sellers et al., 2003) and increases well-being (Latrofa et al., 2009). We thus recommend future research to be directed at identity coping mechanisms that do not involve a dissociation, but rather an integration of women’s gender identity at the workplace.

### Social Identity Processes Among Male STEM Professionals

Contrary to the results for female STEM professionals, no empirical evidence was found that numerical and normative male dominance at work impose barriers to men’s careers; men’s experience of gender identity threat at work was unrelated to these context effects, and gender identity threat did not mediate the relationship between numerical and normative male dominance at work and career perceptions. However, that is not to say that gender identity processes do not play a role for male STEM professionals. For men too, higher gender identification was associated with higher levels of gender identity threat. What’s more, correlational analyses (Table 2) indicated that men’s identification with their gender identity at work was higher when working in the STEM sector relative to outside STEM, and when their work context was composed of a higher majority of men. In addition, when men did feel threatened at work on the basis of their gender identity, this too had a small but significantly negative effect on their career confidence. A crucial question remains what institutional parameters will elicit feelings of gender identity threat among male STEM professionals. Building on recent work, the potential loss of men’s high-status position in STEM in response to implementation of gender quota or pro-diversity programs may form one such identity threat (Dover et al., 2016). This forms an interesting avenue for future research.

Our findings suggest that for men, working in male dominated STEM contexts is inherently connected to their male identity. Recent work demonstrates that masculine professional stereotypes may not only discourage women, but also some men, who feel they are ‘not men enough’ to measure up to the macho stereotypes associated with a professional field (Peters et al., 2014). Peters et al. (2014) demonstrated that this is the case among male commando recruits in the Royal Marine and male surgical trainees in the medical sector. Although the content of masculine stereotypes may be quite different in the STEM sector, in future research a similar investigation in the STEM sector is highly relevant and timely, because even though dropout rates in the STEM sector are highest among women, they are also high among men (about half of men STEM graduates opts for a career outside STEM; Statistics Netherlands, 2016).

First empirical support for the idea that the STEM sector is mostly considered an attractive career option among prototypically masculine STEM graduates (irrespective of their gender) was found in research on STEM students’ professional identity profiles. This work shows that those with a stereotypically “Nerdy” profile (e.g., highly analytical and introverted, values intellectual stimulation, likes computer gaming) identified highest with their professional identity and were most likely to opt for a career in STEM (van Veelen et al., 2018). This suggests that people’s perception about what it means to be a successful professional STEM is quite narrowly defined and masculine. This does not only obstruct women STEM graduates from opting for a career in STEM, but also a lot of men. The STEM sector thus faces the challenge to increase numerical gender diversity in the work force, but also to foster inclusive work climates (Otten and Jansen, 2014) where people with different demographic *and* professional profiles feel accepted and appreciated.

### Limitations and Future Research

We demonstrated that female STEM graduates who work in the STEM sector *and* who work with a majority of men experience the highest levels of gender identity threat. This finding informs us about the social-identity explanations as to why women are more likely to opt for a career outside STEM, or leave the STEM sector at a later point. The fact that male dominance manifests itself on different institutional parameters (i.e., numerical and normative), and that they have unique and joint explanatory power, calls for a further detection and investigation of the combined effects of other institutional parameters that signal male dominance on social identity threat in future research. For example, we may expect that institutional parameters such as organizations’ corporate structure (e.g., flat vs. hierarchical; Morgan, 2014), employment conditions (e.g., contract size, flexible working, leave arrangements (Plantenga and Remery, 2010), or gender diversity policies (Dobbin and Kalev, 2018; Pietri et al., 2018) jointly add to the potency of the work context to form a source of gender identity threat in women’s efforts to build a career.

We assume that working in STEM (vs. non-STEM) serves as a proxy for high (vs. lower) normative male dominance in the work context, and we do so based on prior evidence demonstrating that – particularly in the Netherlands – stereotypically masculine characteristics tend to be positively valued in STEM (Diekman et al., 2010; Leslie et al., 2015; Miller et al., 2015; Storage et al., 2016; Derks et al., 2018) and women’s professional ability tends to be undermined in STEM (e.g., Nosek et al., 2009; Miller et al., 2015). Yet in the current study, we cannot pinpoint the exact nature of normative male dominance, and what specific elements of the STEM professional culture drive women’s higher levels of gender identity threat. Is it the negative gender stereotype that ‘women are worse in math’ (Cheryan et al., 2009), the ‘innate brilliance’ that is attributed to people working in STEM (Leslie et al., 2015), or the ‘performance-driven culture’ in STEM (Bleijenbergh et al., 2012) that cause women to feel more uncertain and negatively judged as a professional in- than outside STEM? In follow-up studies, we suggest to measure STEM professionals’ perceptions of their own work sector (STEM vs. non-STEM) on these specific elements in order to (1) directly test the assumption that higher gender identity threat levels among women working in STEM relative to in other sectors are indeed attributable to a stereotypically higher endorsement of masculine attributes and a lower expectation about women’s ability in STEM work contexts. Relatedly, our holistic approach to differentiate between STEM and non-STEM does not consider that STEM disciplines vary strongly in gender bias and inequality. For example, biology and neurosciences are far more ‘gender-equal’ compared to engineering and physics (Cheryan et al., 2017). Future research would benefit from more fine-grained field studies investigating *what* specific masculine norms in STEM professional cultures make the STEM sector a women-unfriendly place to work, and *where* in different STEM disciplines these gendered norms manifest most strongly.

Because of the cross-sectional nature of the data, claims about causality should be made with caution. While it is quite safe to assume that work context parameters *precede* women’s experience of gender identity threat in that particular context, a reverse causal model in which career attitudes precede gender identity threat could – in theory – be possible, such that *because* the work context negatively affects women’s career confidence and work engagement, it makes them more prone to experience gender identity threats. Nevertheless, a statistical test of this alternative model resulted in poor model fit^6^ and non-significant parameter estimates for both direct and indirect effects, rendering this reverse causal model unlikely. In a similar vein, in the current cross-sectional data we were unable to rule out third variable explanations, for example that individual differences between women who do and do not opt for a career in STEM can explain why women in STEM experience more gender identity threat than women STEM graduates who work outside of STEM. However, we deem it unlikely that those women who are somehow most vulnerable to these settings are the ones who end up choosing them. In any case, future research in the form of experimental or longitudinal designs could offer a more solid method to make causal inferences about contextual causes and career consequences of gender identity threat.

The self-report data in this study may raise concerns about common method bias (Podsakoff et al., 2003)^7^. Yet scale testing (see footnote 7) demonstrated that common method variance was negligible (Podsakoff et al., 2003). In addition, significant moderation effects cannot be artifacts of common method bias (Siemsen et al., 2010). In future research, a multi-source method, for example including objective measures of numerical representation of women and men in the work context and actual turnover rates, promotions and salary raises of professionals working in STEM and non-STEM via personnel records adds further validity to the current study outcomes.

While the ecological validity of our field data is high, we must consider that selection biases are present in our sample. For example, in our sample 77% of the graduates indicated to work in the STEM sector (66% of the women; 81% of the men), while national figures demonstrate that around 30% of all women and 50% of all men STEM graduates opt for a career in STEM (Statistics Netherlands, 2016). A reason for this difference might be that those who decided to stay in STEM after graduation feel more affiliated with their past education and their time at University. Thus, they might be more likely to read emails on their alumni address and respond to requests to participate in research to support STEM students’ career development. Moreover, because this study was set out in the Netherlands – in which gender biases in STEM are relatively high (Miller et al., 2015) – we cannot generalize our findings to other countries. In future research, a cross-cultural comparison can offer valuable insights as to whether levels of gender identity threat in response to working in STEM (vs. non-STEM) differ depending on the endorsement of negative gender stereotypes in STEM on a national level.

In this study, we focused on work context parameters that have *negative* (threatening) consequences for women working in STEM and form barriers to their careers. While this focus is highly valuable to explain why women opt out of STEM, a more solution-driven approach would be to focus on *positive* context parameters that challenge women – and men – working in STEM and form a springboard to their careers. As a first step, recent research demonstrated that the presence of *gender inclusive policies* reduced feelings of gender identity threat among women in engineering (Hall et al., 2018b). Importantly, they demonstrated that these gender inclusive policies reduced feelings of gender identity threat even when the numerical representation of women in the work context was low. As such, even if it is difficult for STEM organizations to attract a higher number of women in their work force because of today’s shortages in highly skilled STEM professionals on the labor market, this should not prevent organizations from advocating gender inclusive norms in order to create an identity-safe working climate, where women want to stay.

## Conclusion

Women enter the STEM sector at lower rates, and leave the STEM sector at higher rates than do men. Taking a social identity approach, this research distinguished between two institutional parameters of male dominance that uniquely but jointly predict female STEM graduates’ experience of gender identity threat at work. Gender identity threat, in turn, served as an explanatory mechanism as to why numerical and normative male dominance in STEM negatively affect women’s career confidence and work engagement. To break this vicious cycle, STEM organizations should aim to improve gender equality at work, both numerically (improving women’s representation) *and* normatively (removing negative gender stereotypes). By removing these contextual barriers, the STEM sector likely becomes a more appealing place to work for a larger, more inclusive group of women and men.

## Data Availability Statement

The data that support the findings of this study are available from the corresponding author, RvV, upon reasonable request.

## Author Contributions

RvV and BD developed the conceptual model and design of the study. ME managed the set-up and roll out of the survey and supervised the RA’s during the data collection processes. RvV performed literature searches, conducted the data analyses, interpreted the results, and wrote the manuscript. BD and ME gave advice at all stages during this process and provided feedback on the manuscript.

## Conflict of Interest Statement

The authors declare that the research was conducted in the absence of any commercial or financial relationships that could be construed as a potential conflict of interest.

## Footnotes

1 The current study variables were part of a larger online survey on professional development in STEM in which we also asked questions about participants’ professional profile, for example with regards to their competences, personality, values and interests as well as additional questions about professional development and learning. Upon request, more information about the complete questionnaire can be obtained from the first author.

2 Our sampling strategy was to obtain a sample size as large and representative for the population as possible (that is, alumni from two STEM educational institutes). This resulted in a sample size of *N* = 807. Because of this strategy, no *a priori* power analysis was conducted. As a general rule of thumb, *N* = 100–150 is considered the minimum sample size for conducting SEM (Ding et al., 1995). Still others advise a larger sample size, for example, *N* = 200 (Boomsma and Hoogland, 2001; Kline, 2015). Simulation studies show that with normally distributed indicator variables and no missing data, a reasonable sample size for a simple SEM model is about *N* = 150 (Muthén and Muthén, 1998/2012). For multi-group modeling, the rule of thumb is 100 observations per group (Kline, 2015). Because the required sample size also depends on the complexity of the model, another widely accepted rule of thumb is 10 observations per variable (Nunnally and Bernstein, 1967). Including covariates and interaction terms, our SEM model consists of 12 variables. This would mean that per gender group, a minimum sample size of *N* = 120 is needed. Thus, based on these rules of thumb, the sample size for both women (*N* = 177) and men (*N* = 630) can be considered large enough for the model we test.

3 *T*-test results with unequal variances assumed were interpreted, as Levine’s test indicated unequal variances between women and men, due to a large difference in group size.

4 We also explored the three-way interaction (work sector × gender ratio × gender identification) and found that it was not significant for either men or women.

5 Note that while these interaction effects were significant for women but not men, the Δχ^2^ tests on the parameter estimates across the gender groups were not significant (gender ratio × gender ID: Δχ^2^[1] = 1.70, *p* = 0.19; work sector × gender ID: Δχ^2^[1] = 2.65, *p* = 0.10).

6 An alternative causal model in which women’s career confidence and work engagement *preceded* gender identity threat resulted in poor model fit (χ^2^[24] = 181.74, *p* < 0.001, χ^2^/df = 7.57, RMSEA = 0.13, SRMR = 0.05. CFI = 0.59, TLI = -0.03).

7 We investigated the presence of common method variance by using Harmans single factor test (Podsakoff et al., 2003), in which all scale items [gender ratio, work sector, gender identification (four items), identity threat (four items), career confidence (six items) and work engagement (two items)] were entered in an unrotated exploratory factor analysis (PCA) with the number of factors constrained to one. Common method bias is assumed be to present when the single factor explains over 50% of variance, yet our resulting factor merely explained 22% of variance in the items.
